# Epidemiology of Radiofrequency Exposure: Ahlbom et al. Respond

**Published:** 2005-03

**Authors:** Anders Ahlbom, Adele Green, Leeka Kheifets, David Savitz, Anthony Swerdlow

**Affiliations:** Instititute of Environmental Medicine, Karolinska Institutet, Stockholm, Sweden, E-mail: anders.ahlbom@imm.ki.se; Queensland Institute of Medical Research, Brisbane Australia; School of Public Health, University of California at Los Angeles, Los Angeles, California; School of Public Health, University of North Carolina at Chapel Hill, Chapel Hill, North Carolina; Institute of Cancer Research, Sutton, Surrey, United Kingdom

We thank Kundi for his comments on our review of the epidemiologic literature on health effects of radiofrequency exposure (Ahlbom et al. 2004). He points out, quite correctly, that our assessment of the literature differs from the one he and colleagues have made in a previous review (Kundi et al. 2004). We do, however, stand by our judgment that the literature we reviewed offers little support for a causal relation between radiofrequency exposure and disease risk. Although quality of research varies, most of the studies we reviewed were methodologically limited and more rigorous studies are needed. It is obviously impossible to tell what more sophisticated research along the lines suggested by Kundi, and also by ourselves, will reveal in the future. We certainly agree that consideration of latency and various types of bias is important and, indeed, we did consider these at some length in our review.

## References

[b1-ehp0113-a0151b] Ahlbom A, Green A, Kheifets L, Savitz D, Swerdlow A (2004). Epidemiology of health effects of radiofrequency exposure. Environ Health Perspect.

[b2-ehp0113-a0151b] Kundi M, Mild K, Hardell L, Mattsson MO (2004). Mobile telephones and cancer—a review of epidemiological evidence. J Toxicol Environ Health B Crit Rev.

